# *Giardia duodenalis* in domestic cats from the US and Canada: Prevalence and zoonotic potential

**DOI:** 10.1371/journal.pone.0358546

**Published:** 2026-10-07

**Authors:** Margaret Wingerd, Anne Kimmerlein, Pablo D. Jimenez Castro, Michelle D. Evason, Jennifer L. Willcox, Cecilia E. Lozoya, Jeffrey Tereski, Kelly Mitchell, Philip J. Bergman, Christian M. Leutenegger, Marie E. Kerl

**Affiliations:** 1 VCA SouthPaws Veterinary Specialists & Emergency Center, Fairfax, Virginia, United States of America; 2 Mars Veterinary Health, Los Angeles, California, United States of America; 3 Antech Diagnostics, Mars Petcare Science & Diagnostics, Loveland, Colorado, United States of America; 4 Grupo de Parasitologia Veterinaria, Universidad Nacional de Colombia, Colombia; Universidad Nacional de Colombia Campus Palmira, COLOMBIA

## Abstract

*Giardia duodenalis* is a flagellated commensal protozoan that can infect both humans and animals. Infection can result in clinical signs such as diarrhea or, more often, the infection can be subclinical. Within the *G. duodenalis* species, there are several major genetic assemblages, A – H. Certain assemblages are species specific (C and D in dogs, F in cats) while assemblages A and B can infect humans as well as other mammals. This retrospective study used data collected from a commercial reference laboratory (Antech Diagnostics, Mars Petcare Science & Diagnostics). Real-time PCR data using fecal qPCR panel (KeyScreen™ GI Parasite PCR) for *Giardia duodenalis* and potential zoonotic assemblages A/B in 157,616 domestic cat stool samples from North America collected between March 8, 2022, to August 31, 2023, was used. Variables including age, sex, altered status, season, and geographic region were assessed to identify associations with cats that were detected for *G. duodenalis* and potential zoonotic assemblages A/B. The overall prevalence of *G. duodenalis* was 6.3% (9,962/157,616) and of those detected, 18.5% (1,840/9,962) were assemblages A/B. Cats that were *G. duodenalis* detected were more likely to be male, intact, less than one-year old, and in the Midwest. In general, lowest detection occurred during the summer months. Zoonotic potential detection was highest in cats aged less than one-year (19.7%; 1,011/5,137). These data highlight the importance of routine fecal screening in domestic cats to detect parasites, particularly those, like *Giardia*, of One Health concern, and provides prevalence, and risk factor information that aids clinical veterinary decision-making.

## Introduction

*Giardia duodenalis* (*Gd*) is a flagellated commensal protozoan that in some instances can become parasitic [1]. Most infections are subclinical in both humans and animals, but on some occasions, these can cause diarrhea and ill thrift [2,3]. *Gd* has a global distribution with a prevalence in humans of 1.4% in the United States (US) [4]. In animals, worldwide prevalence reported in studies varies widely: cattle ranging from 3.7–100%, sheep from 1.5–55.6%, pigs from 0.1–31.1%, dogs from 1.1–56.8%, and cats from 0.2–44.4%. This is due to differing methods of detection, geography, age of the animal, and presence or absence of clinical signs [1,4]. The reported prevalence of *Gd* in cats in the US has ranged from 1.6–10.8% [5–8].

In 2023, there was a proposed taxonomical revision involving *Gd* and its major genetic assemblages with the proposed nomenclature being *G. duodenalis* (A), *G. enterica* (B), *G. canis* (C), *G. lupus* (D), *G. bovis* (E), *G. cati* (F), *G. simoni* (G), and *G. pinnipedis* (H) [9]. However, this change has not been widely accepted. Certain assemblages are species-specific (C and D in dogs, E in hoofed animals, F in cats, G in rodents, and H in pinnipeds), while assemblages A and B can infect humans as well as other mammals [10]. Assemblage A can be further subdivided into four clusters, A-I – A-IV. Previous alloenzyme analysis has found A-I in humans and dogs, A-II only in humans, A-III only in cats, and A-IV in various animals, but not in humans [11]. A more recent genotypic analysis of *Gd* found both assemblages A-I and F in domestic cats in Mississippi and Alabama [12].

Most studies on the prevalence of *Gd* in cats are limited to specific geographic locations, small or very specific populations, with various methods used for detecting the infection [5–8]. The objectives of this retrospective study were to use data collected from anonymized fecal test submissions from cats across the US and Canada, submitted between March 2022 – August 2023, for fecal qPCR (KeyScreen™ GI Parasite PCR, Antech Diagnostics [Mars Petcare Science & Diagnostics, Loveland, CO]) to determine: 1) *Gd* frequency in cats, and to describe, 2) the prevalence of potential *Gd* zoonotic assemblages detected in cats and, 3) any significant associations across different patient characteristics (i.e., signalment, geographic location, season, etc.) related to *Gd* and zoonotic potential detection.

## Materials and methods

### Data collection

The laboratory information management system of a commercial reference laboratory (Antech Diagnostics, Mars Petcare Science & Diagnostics) was searched for fecal qPCR panel (KeyScreen™ GI Parasite PCR) accessions from March 8, 2022, to August 31, 2023. From these data, the subset of results from feline species for *Gd* and *Gd* A/B assemblages associated with zoonotic potential were collected and evaluated. Inclusion criteria consisted of cases with results available for both tests (*Gd* and *Gd* A/B assemblages), samples determined to be from domestic feline species, and geographic location from the US and Canada. Cases not meeting all criteria were excluded.

### Real-time PCR test

The dataset for this study utilized results provided by a commercially available GI parasite molecular test (KeyScreen™ GI Parasite PCR, Antech Diagnostics, Inc., Mars Petcare Science & Diagnostics) [13,14].

### Statistical analysis

Analysis was performed using R statistical software (v4.3.2; R Core Team 2023). Univariate logistic regression was used to explore associations between the prevalence of *Gd* and *Gd* A/B assemblages associated with zoonotic potential and individual risk factors. Age was defined as kittens, from birth up to 1 year; young adult, from 1–6 years; mature adult, from 7–10 years; and senior, 10 years and greater [15]. Seasons were defined based on meteorological seasons on the Northern Hemisphere, with spring including March, April, and May; summer including June, July, and August; fall including September, October, and November; and winter including December, January, and February [16]. Geographically, the US data was divided as per the US Census Bureau into 4 regions: the West, Midwest, South and Northeast, as is commonly described [16–19]. Factors with a significant association (p < 0.05) in univariate analysis were included in multivariate logistic regression model selection. Final multivariate models were selected via a manual backwards elimination procedure using the Likelihood Ratio Test and scientific best judgement. All possible interactions between predictor variables were explored and retained in the final model if statistically significant and/or clinically relevant. Canadian data, data with missing age, sex, or neuter status, and any data resulting in risk factor groups of less than 10 cats were excluded from multivariate regression analysis. Odds ratios in which the 95% CI did not include one were considered statistically significant.

## Results

A total of 157,616 domestic feline fecal submissions from March 8, 2022, to August 31, 2023, were evaluated. Samples were submitted from 49 U.S. states, Washington D.C., and five Canadian provinces (AB, BC, NB, ON, SK). Cats ranged from 1 day to 26 years old and were evenly divided between 49% male (76,618/157,616) and 48% female (74,925/157,616). The majority (71%, 112,193/157,616) of cats were spayed or neutered (Table 2). Fifty-two cat breeds were represented. Table 3 shows the top 10 cat breeds, with the top 4 breeds being domestic shorthair (54.7%, 86,200/157,616), unknown breed (20.8%, 32,770/157,616), domestic long hair (6.6%, 10,356/157,616), and domestic medium hair (6.0%, 9,480/157,616). In total, 9,962 (6.3%) samples were found to have *Gd* detected with 9,514 (95.5%) coming from the US and 448 (4.5%) from Canada. Out of the total 9,962 *Gd* detected samples, 1,840 (18.5%) were found to have detection of assemblages A/B with zoonotic potential, 1,775 (96.4%) from the US and 65 (3.5%) from Canada.

### Giardia duodenalis

A total of 133,914 cats were included in the logistic regression model results for *Gd* (Table 1). Of these, 6.9% of male cats and 5.7% of female cats had *Gd* detected (Table 2). Male cats had 20% greater odds (OR 1.2; 95% CI: 1.15, 1.26) of being detected compared to female cats. Nine percent of intact and 5% of spayed and neutered cats were *Gd* detected. A significant interaction between age and spay/neuter status in the final regression model implied that age modified the effect of spay/neuter status on the odds of a cat being detected for *Gd* (Table 1). For kittens (less than 1-year old), intact cats had 24% lower odds of being *Gd* detected than spayed/neutered cats (OR 0.76; 95% CI: 0.72, 0.81). However, for cats, one-year-old or older, intact cats had greater odds of being *Gd* detected than spayed/neutered cats. Intact young adult cats (1–6 years) had 1.65 (95% CI: 1.49, 1.82) times greater odds and intact senior cats (10 years and greater) had 1.63 (95% CI: 1.03, 2.56) greater odds of being *Gd* detected than spayed/neutered cats. Intact mature adult cats (7–10 years) had a higher rate of *Gd* positivity than spayed/neutered cats of the same age, but this difference was not statistically significant (OR 1.14, 95% CI: 0.67, 1.97).

**Table 1:** Multivariate logistic regression model results for *Giardia duodenalis* detection in 133,914 cats via KeyScreen™ GI Parasite PCR in the United States.

Variable	β	SE	Odds Ratio (OR)	95% CI (OR)		*P*-value
				Lower	Upper	
Sex						
Female	Reference					
Male	0.185	0.023	1.204	1.151	1.259	<0.001
Reproductive Status						
Spayed/Neutered	Reference					
Intact (<1 yr)	−0.276	0.031	0.759	0.715	0.806	<0.001
Intact (1–6 yr)	0.501	0.051	1.651	1.494	1.824	<0.001
Intact (7–10 yr)	0.135	0.277	1.144	0.665	1.968	0.627
Intact (>10 yr)	0.486	0.232	1.625	1.031	2.561	0.036
Age						
<1 yr (Intact)	1.689	0.227	5.413	3.473	8.440	<0.001
<1 yr (S/N)	2.450	0.059	11.590	10.319	13.014	<0.001
1-6 yr (Intact)	1.610	0.230	5.000	3.184	7.854	<0.001
1-6 yr (S/N)	1.594	0.059	4.924	4.389	5.523	<0.001
7-10 yr (Intact)	0.091	0.352	1.095	0.549	2.181	0.797
7-10 yr (S/N)	0.442	0.082	1.555	1.323	1.826	<0.001
>10 yr	Reference					
Season						
Summer	Reference					
Winter	0.678	0.031	1.970	1.852	2.094	<0.001
Spring	0.623	0.030	1.865	1.757	1.979	<0.001
Fall	0.549	0.034	1.731	1.618	1.851	<0.001
Region						
South	Reference					
Midwest	0.308	0.033	1.361	1.276	1.452	<0.001
Northeast	0.053	0.030	1.054	0.994	1.118	0.080
West	−0.047	0.031	0.954	0.897	1.014	0.134
S/N, spayed or neutered						

**Table 2:** *Giardia duodenalis* and *G. duodenalis* A/B assemblages with zoonotic potential detection in 149,796 cats tested via KeyScreen™ GI Parasite PCR in the United States.

Characteristic	*Giardia duodenalis* N = 9514		*G. duodenalis* A/B assemblages N = 1775	
	No. (%)	*P*-value*	No. (%)	*P* -value*
Sex		<0.001		0.065
Female	4054 (5.7)		720 (17.8)	
Male	5001 (6.9)		964 (19.3)	
Unknown^±^	459 (7.7)		91 (19.8)	
Reproductive Status		<0.001		0.048
Spayed/Neutered	5622 (5.3)		1010 (18.8)	
Intact	3433 (9.2)		674 (19.6)	
Unknown^±^	459 (7.7)		91 (19.8)	
Age		<0.001		0.003
<1 yr	4875 (10.7)		970 (19.9)	
1-6 yr	2984 (6.3)		535 (17.9)	
6-10 yr	286 (2.0)		45 (15.7)	
>10 yr	362 (1.3)		49 (13.5)	
Unknown^±^	1007 (7.1)		176 (17.5)	
Season		<0.001		0.003
Summer	2847 (4.6)		544 (19.1)	
Winter	2339 (8.1)		478 (20.4)	
Spring	2635 (7.1)		433 (16.4)	
Fall	1693 (7.7)		320 (18.9)	
Region		<0.001		<0.001
South	3099 (5.9)		505 (16.3)	
Midwest	1792 (7.8)		304 (17.0)	
Northeast	2611 (6.4)		563 (21.6)	
West	2012 (6.1)		403 (20.0)	

*Univariate logistic regression.

±not included in statistical analysis.

As age increased, the proportion of *Gd* detected decreased (Fig 1; Table 2), with kittens having the highest rate of detection (10.7%) and senior cats having the lowest (1.3%). In kittens, neutered cats had 11.59 (95% CI: 10.32, 13.02) times greater odds and intact cats had 5.41 (95% CI: 3.47, 8.44) times greater odds of being *Gd* detected than senior cats. In young adult cats, neuter status did not have a significant modifying effect on age (P = 0.938), with intact cats having 5.00 (95% CI: 3.18, 7.85) times greater and neutered cats having 4.92 (95% CI: 4.39, 5.52) times greater odd of being *Gd* detected than senior cats. Neutered mature adult cats had 1.55 (95% CI: 1.32, 1.83) times greater odds of being *Gd* detected than senior cats. However, intact mature adult cats did not have statistically different odds of *Gd* detection than senior cats (OR 1.09; 95% CI: 0.55, 2.18).

**Fig 1:**
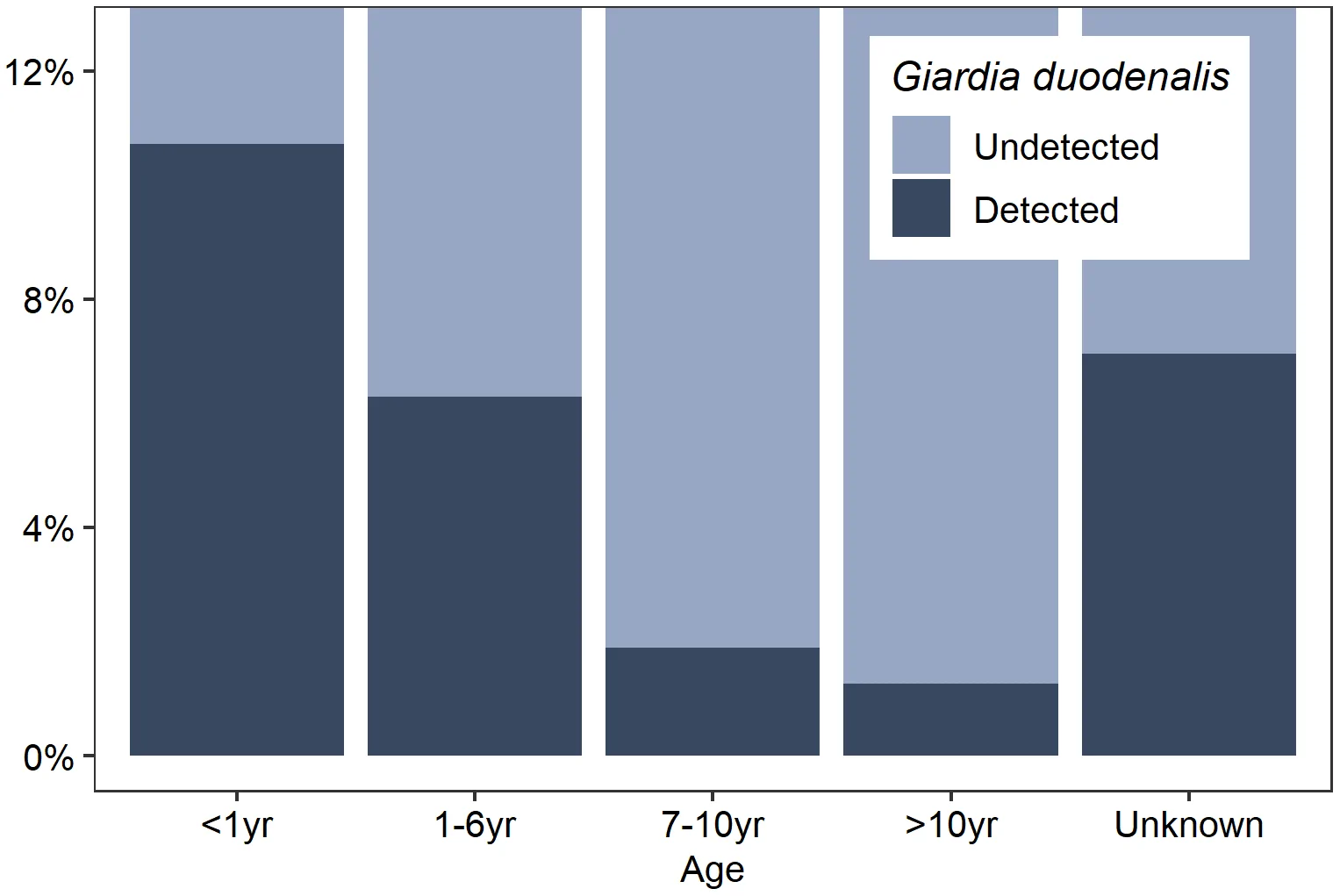
Prevalence of *Giardia duodenalis* by age in 157, 617 cats tested for *Giardia duodenalis* via qPCR.

Summer months had the lowest *Gd* detection with 4.6% of cats (Fig 2; Table 2). Positivity was similar across spring, fall, and winter (7.1%, 7.7%, 8.1% respectively). In spring, cats had 1.86 (5% CI: 1.76, 1.98) times, in fall 1.73 (95% CI: 1.62, 1.85) times, and in winter 1.97 (95% CI: 1.85, 2.09) times greater odds of being *Gd* detected than in summer. The Midwest had the highest detection of *Gd*, with 7.8% of cats showing *Gd* detected, followed by the Northeast (6.4%), West (6.1%), and South (5.9%) (Fig 3). Cats in the Midwest had 1.36 (95% CI: 1.28, 1.46) times greater odds of being *Gd* detected than cats in the South. However, there was no significant difference between *Gd* positivity in cats in the Northeast (P = 0.134) and West (P = 0.080) compared to the South.

**Fig 2:**
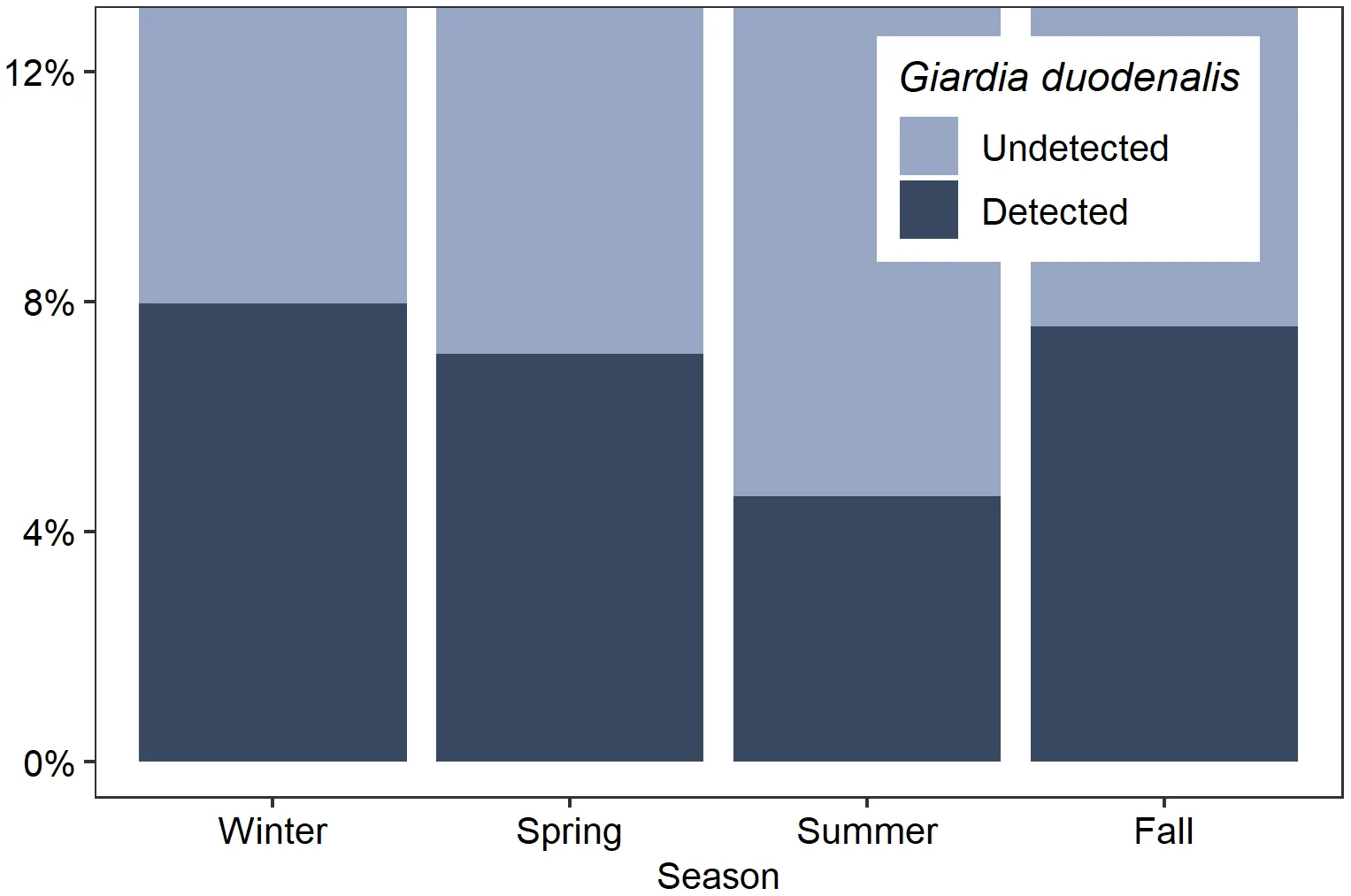
Prevalence of *Giardia duodenalis* by season in 157, 617 cats tested for *Giardia duodenalis* via qPCR.

**Fig 3:**
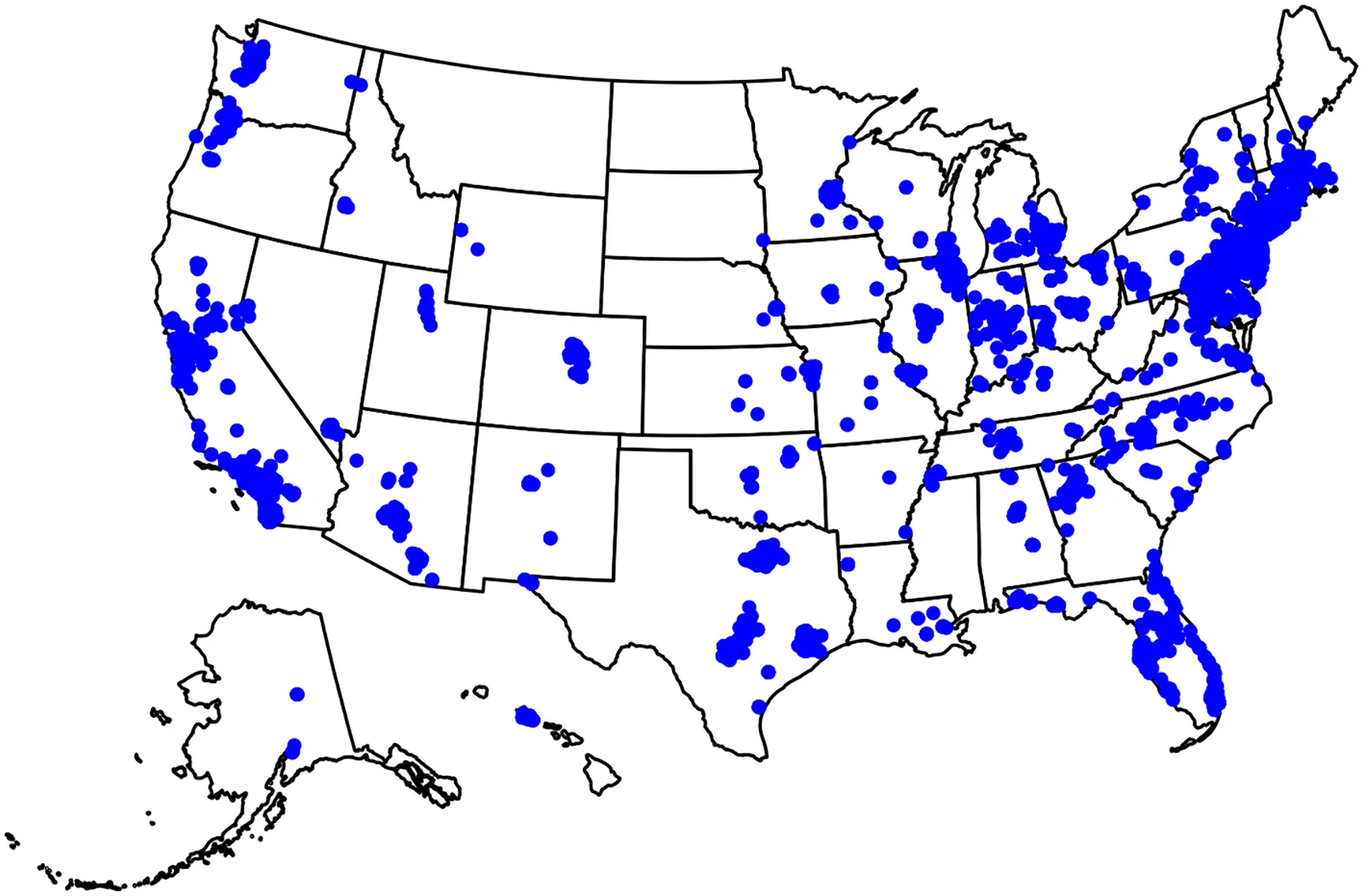
Map of *Giardia duodenalis* detected in cats from United States.

### *G. duodenalis* A/B Assemblages with zoonotic potential

A total of 8,404 cats that were *Gd* detected were included in the logistic regression model results for A/B assemblages with zoonotic potential (A/B) detection (Table 3). Of *Gd* detected cats, a greater proportion of males (19.3%) than females (17.8%) were A/B detected (Table 2), but this difference was not statistically significant (P = 0.078; Table 3). Likewise, a higher proportion of *Gd* detected intact cats (19.6%) than *Gd* detected spayed/neutered cats (18.0%) were A/B detected, but this difference was also not statistically significant (P = 0.440).

**Table 3:** Multivariate logistic regression model results for *Giardia duodenalis* A/B assemblages with zoonotic potential in 8404 cats where *G. duodenalis* was detected via KeyScreen™ GI Parasite PCR in the United States.

Variable	β	SE	Odds Ratio (95% CI)	*P*-value
Sex				
Female	Reference			
Male	0.100	0.057	1.105 (0.989, 1.236)	0.078
Reproductive Status				
Spayed/Neutered	Reference			
Intact	0.049	0.063	1.050 (0.927, 1.189)	0.440
Age				
<1 yr	0.389	0.162	1.475 (1.084, 2.047)	0.016
1-6 yr	0.284	0.162	1.329 (0.976, 1.844)	0.079
7-10 yr	0.156	0.224	1.168 (0.751, 1.814)	0.488
>10 yr	Reference			
Season				
Spring	Reference			
Summer	0.191	0.076	1.210 (1.042, 1.406)	0.013
Winter	0.265	0.078	1.303 (1.118, 1.520)	<0.001
Fall	0.119	0.088	1.127 (0.947, 1.338)	0.177
Region				
South	Reference			
Midwest	0.049	0.084	1.050 (0.890, 1.238)	0.562
Northeast	0.403	0.073	1.496 (1.296, 1.726)	<0.001
West	0.294	0.077	1.342 (1.153 1.561)	<0.001

As age increased, the proportion of *Gd* detected cats that were A/B detected decreased (Fig 4; Table 3). Kittens had the highest proportion of A/B positivity (19.9%), and senior cats had the lowest (13.5%). Kittens had 1.47 (95% CI: 1.08, 2.05) times greater odds of being A/B detected than senior cats. However, there was not a statistically significant difference in odds of A/B positivity between young adult (P = 0.079) or mature adult cats (P = 0.488) and senior cats (Table 3).

**Fig 4:**
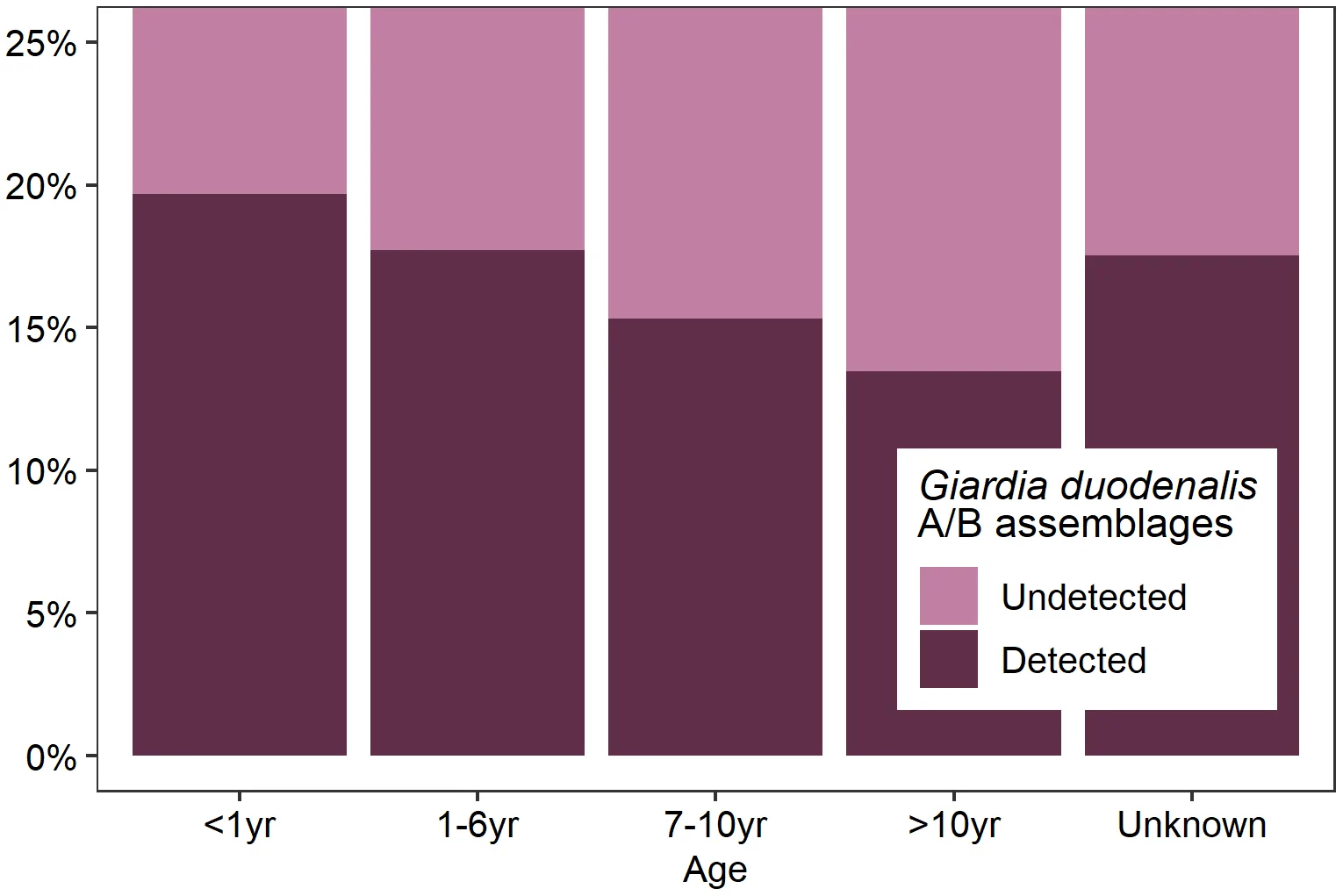
Proportion of 9.962 *Giardia duodenalis* detected cats via qPCR that had zoonotic *Giardia* assemblages by age group.

Winter had the highest proportion of *Gd* detected cats that were A/B detected (20.4%), followed by summer (19.1%), fall (18.9%), and spring (16.4%) (Fig 5). In the winter, cats had 1.30 (95% CI: 1.12, 1.52) times and in the summer, 1.21 (95% CI: 1.04, 1.41) times greater odds of being A/B detected than in the spring. The odds of A/B positivity were not significantly different in fall versus spring (P = 0.177). Cats in the Northeast had the highest A/B detection (21.6%), followed by the West (20.0%), the Midwest (17.0%), and the South (16.3%) (Fig 6). Cats in the Northeast had 1.50 (95% CI: 1.30, 1.73) times, and cats in the West had 1.34 (95% CI: 1.15, 1.56) times greater odds of being A/B detected than cats in the South. There was not a significant difference in the proportion of *Gd* cats that were A/B detected in the Midwest compared to the South (P = 0.562).

**Fig 5:**
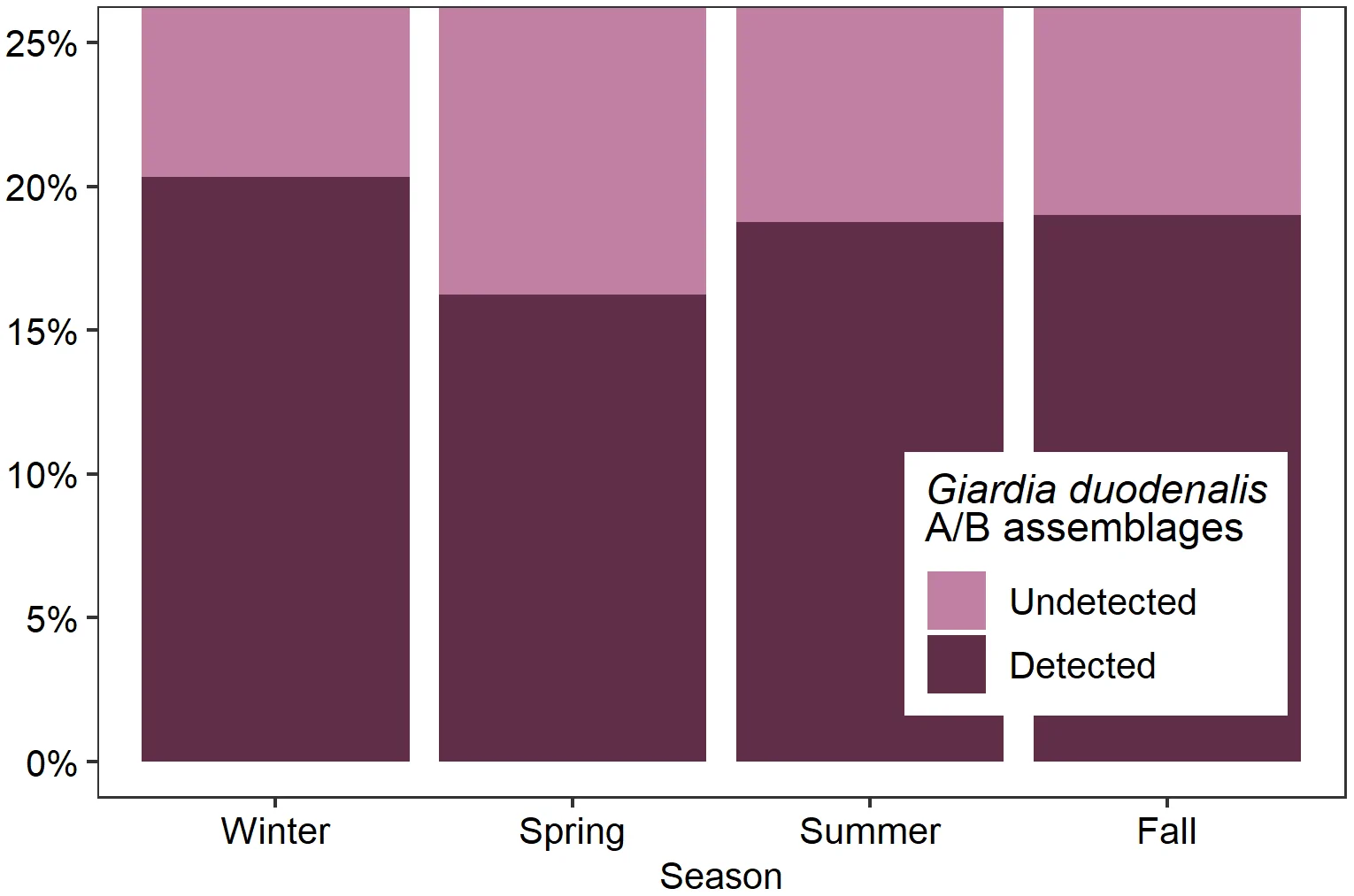
Proportion of 9.962 *Giardia duodenalis* detected cats via qPCR that had zoonotic *Giardia* assemblages by season.

**Fig 6:**
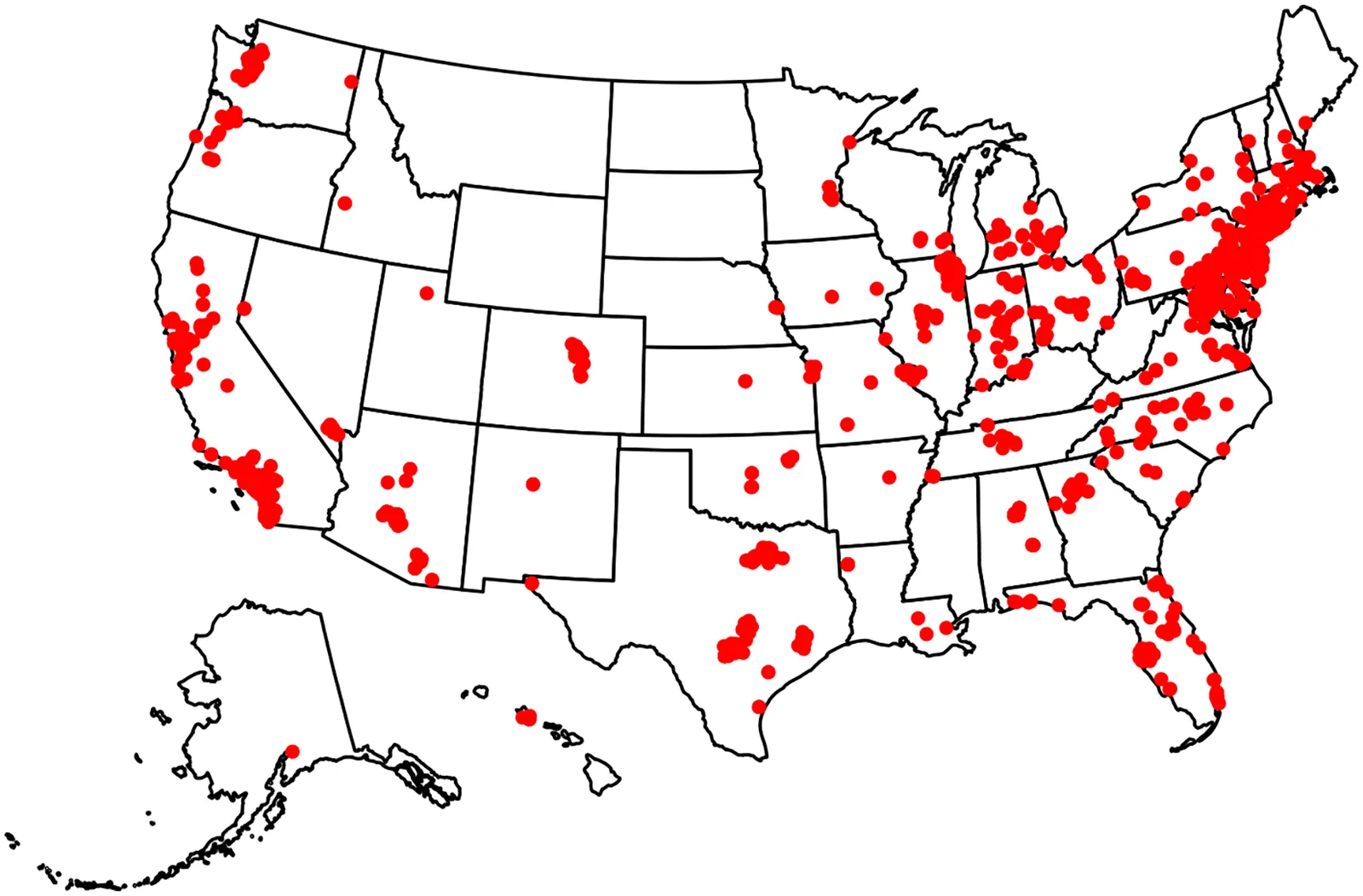
Map of Zoonotic potential *Giardia duodenalis* detected in cats from the United States.

## Discussion

To the authors’ knowledge, this study describes the largest domestic feline population from the US and Canada tested using a molecular qPCR diagnostic for *Gd* and assemblages with zoonotic potential A/B. Of 157, 616 total samples, 6.3% were detected for *Gd*, which is on the higher end, but within the range previously published for the US using alternate methods of detection like centrifugal flotation and/or ELISA [5–8,20].

A previous meta-analysis showed that a majority of cats with *Gd* have assemblage F (55.8%), followed by A (38.7%), and only a small percentage have assemblage B (2.8%) [21]. In the US, previous genotypic analyses of *Gd* in cats have shown the percentage of cats with assemblage A to range from 21.3–35.3% [12,22,23]. Of the *Gd* detected samples in our study, 18.5% were found to have the potential zoonotic assemblages A/B, which was lower compared to these previous studies [12,22,23]. This is likely due to the small population sizes and specific geographic locations (eastern Mississippi and northwestern Alabama; Virginia; Colorado) of these other studies [12,22,23]. Due to the much broader geographic range, encompassing most of the US and portions of Canada, plus the much larger sample population size, our data likely provides a better prevalence estimate of cats with *Gd* and *Gd* with zoonotic potential.

The test method to identify *Gd* assemblages for our study was based on a bg gene real-time PCR (qPCR). This method was adapted from previously published protocols and has been shown to identify assemblages A and B, including human specific sub-assemblages of A and B confirmed by multi-locus sequencing [24–26]. While cats harbor mostly assemblage F, some *Gd* cases do represent assemblage A and with a lower frequency also B [27]. Both assemblages A and B in cats have been shown to contain human zoonotic sub-assemblages [26].

Male cats were found to have much higher odds of *Gd* infection compared to females and intact cats had higher odds of *Gd* infection compared to spayed/neutered cats, which is in agreement with other studies [28]. The higher odds in males and intact cats may be due to exposure differences because of housing differences (i.e., indoor versus outdoor), increased roaming behavior, which was not assessed in this study, or differences in immune competency.

In general, we found that increasing age reduces the odds of *Gd* infection. This is similar to previous studies which reported younger cats to be more susceptible to infection [29–31]. There are likely several factors that contribute to this including improved acquired immunity, decreased exposure due to changes in housing and activity, and decreasing frequency of *Gd* diagnostics [32]. The immune response to *Gd* infection in cats is poorly understood, but based on studies in other animals, it is presumed to be due to cellular immunity and IgA response [3]. Several studies have documented an increase in IgA in aging animals, which could improve immunologic reaction against *Gd* with increasing age [33,34].

Our results indicate that the spay/neuter status of cats influences the odds of *Gd* detection differently in different age groups. Intact kittens had lower odds for *Gd* than neutered kittens, but intact adult and senior cats have higher odds for *Gd* than those spayed and neutered. The apparent protective nature of remaining intact as a kitten may be due, at least in part, to lifestyle factors that we were unable to measure or control for in our study. For example, it may be that spayed and neutered kittens in our study represent a population of cats acquired from shelter or rescue settings, where these kittens are often spayed/neutered at a young age, sometimes as early as 8 weeks old. Cats acquired from other settings (found, from friends or neighbors, or from one’s own queen, etc.) may not be spayed and neutered until closer to a year old in many cases. There is ample evidence to support that cats housed in shelter and rescue settings experience stress, suppressing their immune response, and increasing their susceptibility to infectious diseases [35,36]. Therefore, it is plausible that both exposure to a shelter setting and the effect of experiencing anesthesia and surgery with an immature immune system may make spayed/neutered kittens more susceptible to *Gd*.

*Gd* cysts can survive for a prolonged time in cool and moist environments [37]. This could explain the lower infection rate in summer in general, as summer tends to be warm and dry, and cysts are less likely to survive. In people, *Gd* infection has been reported with a higher frequency in northern US, with higher overall prevalence in the Northeast and West suspected due to regional differences in transmission of *Gd* and differences in reporting [38,39]. This is consistent with our findings of higher *Gd* detection in cats in the Northeast and West compared to the South.

Male and female cats had similar detection of potential zoonotic assemblages, regardless of their spay/neuter status. Although intact males are more likely to have *Gd* detected, this subpopulation is expected to have decreased exposure to humans due to their lifestyle and therefore less likely to have zoonotic potential as detection of assemblages A/B in dogs and cats may be caused by coprophagy and/or spill-back from human contact [4,40]. Zoonotic potential was highest in cats aged less than one-year (19.3%) compared to other age classes. Zoonotic potential was also found to be the lowest during spring, but only significantly lower compared to winter and summer (Table 3.) compared to other seasons, and highest in the Midwest compared to other regions. This is likely due to the overall *Gd* detection being highest in these groups.

The main limitations of this study are related to its retrospective nature. There was limited clinical information with factors such as indoor/outdoor status, presentation, husbandry, clinical signs, travel, or medication history unavailable for this population. Future prospective studies will be needed to determine the importance, if any, of these characteristics.

Given the ability of *Gd* to infect a wide variety of hosts including people and animals, this protozoan can present a public health concern [27,41]. Based on our results, about 18% of cats in the US and Canada infected with *Gd* have potential zoonotic assemblages. This highlights the need for routine fecal screening in domestic cats, especially in cats at a higher risk for *Gd* or those living in households with young children, the elderly, and immunocompromised people. Further investigation should be aimed at determining why certain cat breeds may be more susceptible to potential zoonotic assemblages of *Giardia.* In addition, epidemiological studies using subtyping tools will be able to determine the extent of spillover and spillback (i.e., reverse zoonosis) between pet cats and their owners.

## Supporting information

S1 File Dataset. (CSV)

## Abbreviations

Gd: Giardia duodenalis
